# Association between circulating SerpinB1 levels and insulin sensitivity in Japanese with type 2 diabetes: A single-center, cross-sectional, observational study

**DOI:** 10.1371/journal.pone.0276915

**Published:** 2022-11-04

**Authors:** Mayu Kyohara, Daisuke Miyashita, Ryota Inoue, Kuniyuki Nishiyama, Takahiro Tsuno, Tomoko Okuyama, Yu Togashi, Yasuo Terauchi, Jun Shirakawa

**Affiliations:** 1 Department of Endocrinology and Metabolism, Graduate School of Medicine, Yokohama City University, Yokohama, Japan; 2 Laboratory of Diabetes and Metabolic Disorders, Institute for Molecular and Cellular Regulation (IMCR), Gunma University, Maebashi, Japan; Medical University of Vienna, AUSTRIA

## Abstract

Plasma and liver SerpinB1 levels are elevated in mice with insulin resistance and promote β-cell proliferation in human islets. We measured serum SerpinB1 levels in Japanese subjects with or without type 2 diabetes (T2DM). We enrolled 12 normal glucose tolerance (NGT) and 51 T2DM subjects. There was no difference in serum SerpinB1 levels between the 2 groups (T2DM, 1.3 ± 0.9 ng/mL *vs*. NGT, 1.8 ± 1.7 ng/mL; *P* = 0.146). After adjusting for age and sex, the serum SerpinB1 levels were positively correlated with HOMA2-%S (β = 0.319, *P* = 0.036), and negatively correlated with fasting blood glucose (β = -0.365, *P* = 0.010), total cholesterol (β = -0.396, *P* = 0.006), low-density lipoprotein (LDL) cholesterol (β = -0.411, *P* = 0.004), triglycerides (β = -0.321, *P* = 0.026), and γGTP (β = -0.322, *P* = 0.026) in subjects with T2DM. Thus, circulating SerpinB1 is possibly associated with insulin sensitivity and better blood glucose level in Japanese subjects with T2DM.

**Trial registration:** UMIN Clinical Trials Registry, UMIN000020453.

## Introduction

SerpinB1 (serine protease inhibitor family B member 1), a member of the serine protease inhibitor (SERPIN) family, is also a leukocyte elastase inhibitor and is broadly expressed and abundant in the cytoplasm of neutrophils [1]. El Ouaamari *et al*. [2] previously reported that liver and plasma SerpinB1 levels were elevated in liver-specific insulin receptor knockout mice, and SerpinB1 promoted β-cell proliferation in multiple species, including humans, through humoral interaction with the liver. They also reported that plasma SerpinB1 levels positively correlated with insulin resistance using the composite insulin sensitivity index (CISI) in individuals who had risk factor(s) for type 2 diabetes (T2DM) [2]. There were also several reports on serum SerpinB1 levels in T2DM subjects. Takebayashi *et al*. [3] reported that serum SerpinB1 levels were higher in T2DM subjects than in healthy controls and were negatively correlated with serum low-density lipoprotein cholesterol (LDL-Chol) levels. Using the insulin sensitivity index (SI), Glicksman *et al*. [4] reported that plasma SerpinB1 levels were positively correlated with insulin sensitivity, but not β-cell function in nondiabetic subjects. Kamal *et al*. [5] showed that serum SerpinB1 levels were significantly lower in T2DM subjects than in nondiabetic subjects, positively associated with C-peptide and homeostasis model assessment for β-cell function (HOMA2-%β), and negatively associated with fasting blood glucose (FBG), HbA1c, total cholesterol (T-Chol), and triglycerides (TG). Kassem *et al*. [6] demonstrated that *SERPINB1* gene single nucleotide polymorphisms (SNPs) were associated with glycemic control and β-cell function in T2DM subjects. Altogether, the significance of circulating SerpinB1 in humans within the context of glucose metabolism remains controversial.

In this study, we measured the levels of serum SerpinB1 and analyzed the correlation with metabolic parameters to determine the significance of serum SerpinB1 as a biomarker for glucose metabolism in Japanese subjects.

## Materials and methods

### Ethics statement

This study was registered in the UMIN Clinical Trials Registry (UMIN000020453). All the subjects agreed to participate in this study by providing written informed consent in accordance with the ethical committee regulations of the Yokohama City University Hospital (B151101011). This study conforms to the provisions of the Declaration of Helsinki.

### Patient selection

We collected blood samples from 51 T2DM subjects and 12 normal glucose tolerance (NGT) subjects with obesity and/or endocrine disease who had been admitted to Yokohama City University Hospital between January 2016 and June 2017. All the patients were between 20 to 70 years of age. Subjects with liver cirrhosis, renal dysfunction with a baseline estimated glomerular filtration rate (eGFR) of <30 mL min^-1^ 1.73 m^-2^, severe ketosis or diabetic coma, severe infections, cancer, a perioperative status, or severe trauma were excluded in this study. The use of anti-diabetic medicine in T2DM subjects is shown in **S1 Table in S1 File**.

### Measurement of biochemical parameters

Blood samples were collected following an overnight fast and more than 12 hours after the last dose of anti-diabetic medicine. All the biochemical parameters, including FBG, fasting serum insulin, C-peptide, HbA1c, T-Chol, LDL-Chol, high-density lipoprotein cholesterol (HDL-Chol), TG, aspartate aminotransferase (AST), alanine aminotransferase (ALT), γ-glutamyl transpeptidase (γGTP), and serum creatinine levels were concurrently measured using standard methods at the clinical laboratory of Yokohama City University Hospital. Serum insulin and C-peptide levels were determined by the electrochemiluminescence immunoassay (ECLIA) method (ECLusys® insulin kit, ECLusys® C-peptide kit; Roche Diagnostics). HbA1c was measured using high-performance liquid chromatography and was calculated according to the National Glycohemoglobin Standardization Program (NGSP). LDL-Chol was measured using a direct method. The eGFR was calculated as follows: eGFR (mL/min/1.73 m^2^) = 194 × serum creatinine^−1.094^ (mg/dL) × Age^−0.287^ (years) (for men), and eGFR (mL/min/1.73 m^2^) = 194 × serum creatinine^−1.094^ (mg/dL) × Age^−0.287^ (years) × 0.739 (for women) [7]. Homeostasis model assessment of insulin resistance (HOMA-IR), a marker of insulin resistance [8], was calculated as fasting insulin (μIU/mL) × FBG (mg/dL)/405. The serum insulin and HOMA-IR data for T2DM subjects treated with insulin were excluded from the analyses. HOMA2-%β, a marker of β-cell function, and homeostasis model assessment for insulin sensitivity (HOMA2-%S), a marker of insulin sensitivity, were calculated via a HOMA Calculator using FBG (mg/dL) and fasting C-peptide (ng/mL) (https://www.dtu.ox.ac.uk/homacalculator/download.php). The C-peptide immunoreactivity (CPR) index, an index of the insulin secretion capacity [9], was calculated as fasting C-peptide (ng/mL)/FBG (mg/dL) × 100. The quantitative insulin sensitivity check index (QUICKI), an index of insulin sensitivity, was calculated as 1/[log(fasting serum insulin (μIU/mL)) + log(FBG (mg/dL))] [10]. The fibrosis index based on four factors (FIB‐4 index) was calculated using Sterling’s formula [11]. We measured the serum SerpinB1 levels using ELISA (LS-F13273; LifeSpan BioSciences, Inc., Seattle, W, USA). The intra- and inter-assay coefficients of variation were <4.7% and <9.2%. The data for serum insulin and QUICKI in T2DM subjects treated with insulin were excluded from all of the analyses. In this study, there are some missing data because they are decided not to measure in the context of clinical significance.

### Statistical analysis

Data were reported as the means ± SD. Statistical analyses using Student’s t-test, chi-square test, post hoc power analysis, analysis of co-variance (ANCOVA), Anderson-Darling test, and univariate and multivariate linear regression analyses were conducted with JMP statistical software (JMP Pro version 15.0.0; SAS Institute, Cary, NC, USA). Data were tested for a normal distribution using the Anderson-Darling test. Variables that were not normally distributed were log-transformed for both the univariate and multivariate linear regression analyses (*variables in tables). The raw data for the variable of age followed a roughly normal distribution, and the log-transformation increased the kurtosis and skewness of data distribution. Consequently, we analyzed the variable of age as raw data in the univariate and multivariate linear regression analyses. Significance was defined if the *P* value was <0.05.

## Results

We determined the serum SerpinB1 levels in sera from 51 T2DM and 12 NGT subjects using ELISA (**Table 1**). There was no difference in serum SerpinB1 levels between the 2 groups (T2DM, 1.3 ± 0.9 ng/mL *vs*. NGT, 1.8 ± 1.7 ng/mL; *P* = 0.146). A post hoc power calculation indicated that the statistical power of the results for serum SerpinB1 levels between NGT and T2DM subjects was 22%. Thus, the sample size was insufficient to lead to definitive conclusions for the difference in serum SepinB1 levels among 2 groups. Mean estimated duration of diabetes was 9.3 ± 8.2 years. The T2DM subjects had significantly higher body mass index (BMI) (27.8 ± 6.9 *vs*. 22.1 ± 4.2; *P* = 0.008), waist circumference (95.9 ± 13.9 cm *vs*. 83.0 ± 12.4 cm; *P* = 0.006), FBG (163.5 ± 53.2 mg/dL *vs*. 97.8 ± 16.3 mg/dL; *P* <0.001), fasting serum insulin (12.6 ± 9.4 μIU/mL *vs*. 5.5 ± 2.8 μIU/mL; *P* = 0.016), HOMA-IR (4.8 ± 3.1 *vs*. 1.3 ± 0.9; *P* <0.001), and HbA1c levels (9.3 ± 2.5% (78.4 ± 27.6 mmol/mol) *vs*. 5.5 ± 0.3% (36.5 ± 3.0 mmol/mol); *P* <0.001) and a lower HOMA2-%S (57.6 ± 37.0 *vs*. 89.0 ± 37.9; *P* = 0.015) and QUICKI (0.3 ± 0.0 *vs*. 0.4 ± 0.0; *P* <0.001), both of which were indices of insulin sensitivity, compared with the NGT subjects. After adjustment for BMI using analysis of co-variance (ANCOVA), the significant differences in waist circumference, fasting serum insulin, and HOMA2-%S between NGT and T2DM subjects were disappeared, whereas the increase in FBG, HOMA-IR, and HbA1c and the decrease in QUICKI in T2DM subjects compared to NGT subjects remained significant (*P* <0.05). C-peptide levels were comparable among 2 groups before and after the adjustment for BMI. Furthermore, the adjustment for BMI demonstrated a lower HOMA2-%β in T2DM subjects compared to NGT subjects (*P* = 0.001) (**Table 1**). No differences in serum cholesterol level, or hepatic/renal function were observed between 2 groups (**Table 1**).

**Table 1:** Serum SerpinB1 levels and metabolic characteristics of T2DM and NGT subjects.

	NGT	T2DM	*P* value; NGT *vs*. T2DM	*P* value; NGT *vs*. T2DM (After adjustment for BMI)
	value (n)	value (n)		
Serum SerpinB1 (ng/mL)	1.8 ± 1.7 (12)	1.3 ± 0.9 (51)	0.146	0.093
Age (years)	48.3 ± 16.5 (12)	54.2 ± 12.5 (51)	0.167	0.089
No. men/women	7/5	28/23	0.833	0.565
Estimated duration of diabetes (years)	-	9.3 ± 8.2 (43)	-	-
BMI	22.1 ± 4.2 (12)	27.8 ± 6.9 (51)	0.008	-
Waist circumference (cm)	83.0 ± 12.4 (11)	95.9 ± 13.9 (48)	0.006	0.126
Systolic blood pressure (mmHg)	126.3 ± 20.4 (12)	131.8 ± 19.0 (51)	0.381	0.518
Diastolic blood pressure (mmHg)	78.8 ± 17.3 (12)	79.9 ± 15.4 (51)	0.836	0.929
Fasting blood glucose (mg/dL)	97.8 ± 16.3 (11)	163.5 ± 53.2 (50)	<0.001	<0.001
Fasting serum insulin (μIU/mL)	5.5 ± 2.8 (12)	12.6 ± 9.4 (28)	0.016	0.290
C-peptide (ng/mL)	1.8 ± 0.7 (12)	2.6 ± 1.6 (50)	0.095	0.805
HOMA-IR	1.3 ± 0.9 (11)	4.8 ± 3.1 (28)	<0.001	0.011
HOMA2-%β	97.2 ± 22.8 (11)	64.8 ± 55.5 (47)	0.064	0.001
CPR index	1.8 ± 0.5 (11)	1.8 ± 1.5 (50)	0.987	0.102
HOMA2-%S	89.0 ± 37.9 (11)	57.6 ± 37.0 (47)	0.015	0.086
QUICKI	0.4 ± 0.0 (11)	0.3 ± 0.0 (28)	<0.001	0.001
HbA1c (%)	5.5 ± 0.3 (10)	9.3 ± 2.5(45)	<0.001	<0.001
HbA1c (mmol/mol)	36.5 ± 3.0 (10)	78.4 ± 27.6 (45)	<0.001	<0.001
Total cholesterol (mg/dL)	191.0 ± 28.3 (12)	196.2 ± 42.0 (51)	0.685	0.756
LDL cholesterol (mg/dL)	116.8 ± 25.0 (12)	118.2 ± 34.7 (51)	0.892	0.992
HDL cholesterol (mg/dL)	59.3 ± 20.1 (12)	58.0 ± 56.4 (51)	0.940	0.753
Triglycerides (mg/dL)	119.0 ± 103.8 (12)	179.3 ± 107.5 (51)	0.083	0.339
AST (IU/L)	23.1 ± 12.7 (12)	33.5 ± 43.0 (51)	0.413	0.524
ALT (IU/L)	24.3 ± 22.7 (12)	35.3 ± 34.6 (51)	0.298	0.605
γGTP (IU/L)	47.0 ± 68.7 (12)	77.8 ± 153.9 (51)	0.502	0.521
FIB-4 index	0.9 ± 0.3 (12)	1.6 ± 2.4 (48)	0.349	0.259
eGFR (mL/min)	79.5 ± 19.8 (12)	79.9 ± 25.8 (51)	0.968	0.538
Urine albumin/Cre (mg/gCre)	8.9 ± 11.3 (10)	142.0 ± 497.0 (51)	0.403	0.739

AST, Aspartate aminotransferase; ALT, Alanine aminotransferase; BMI, Body mass index; CPR index, C-peptide immunoreactivity index; eGFR, Estimated glomerular filtration rate; FIB-4 index, fibrosis index based on four factors; γGTP, γ-glutamyl transpeptidase; HOMA-IR, Homeostasis model assessment of insulin resistance; HOMA2-%β, Homeostasis model assessment for β-cell function; HOMA2-%S, Homeostasis model assessment for insulin sensitivity; HDL cholesterol, High-density lipoprotein cholesterol; LDL cholesterol, Low-density lipoprotein cholesterol; NGT, Normal glucose tolerance; T2DM, Type 2 diabetes; QUICKI, Quantitative insulin sensitivity check index

The differences between NGT and T2DM groups were tested by Student’s t-test and chi-square test, or analysis of co-variance (ANCOVA) to adjust for BMI. Data are shown as mean ± SD.

To assess the effects of glycemic control, the HbA1c levels were categorized into quartiles in T2DM subjects; first quartile (Q1): HbA1c ≤7.5%, second quartile (Q2): 7.5% < HbA1c ≤8.6%, third quartile (Q3): 8.6% < HbA1c <10.3%, and fourth quartile (Q4): 10.3% ≤ HbA1c. Then, serum SerpinB1 levels were not different between controlled (Q1) and uncontrolled (Q4) T2DM subjects (*P* = 0.487) (**S2 Table in S1 File**). All quartiles also did not show changes in SerpinB1 levels compared to NGT subjects (*P* = 0.170–0.726) (**S2 Table in S1 File**). Hence, it seems likely that serum SerpinB1 levels are not associated with glycemic control.

The correlations between the serum SerpinB1 levels and metabolic parameters in T2DM were assessed using univariate linear regression analyses. In the T2DM subjects, the serum SerpinB1 levels were negatively correlated with FBG (β = -0.361, *P* = 0.010), T-Chol (β = -0.336, *P* = 0.016), LDL-Chol (β = -0.342, *P* = 0.014), and γGTP (β = -0.287, *P* = 0.042) (**Table 2**).

**Table 2:** Univariate linear regression analyses for serum SerpinB1 levels in T2DM subjects.

	β	*P*
Age (years)	-0.179	0.210
Sex	-0.006	0.966
Estimated duration of diabetes (years) *	0.105	0.503
BMI *	0.134	0.350
Waist circumference (cm) *	0.036	0.810
Systolic blood pressure (mmHg)	0.031	0.828
Diastolic blood pressure (mmHg)	-0.158	0.270
Fasting blood glucose (mg/dL) *	-0.361	0.010
Fasting serum insulin (μIU/mL) *	-0.049	0.804
C-peptide (ng/mL) *	-0.106	0.466
HOMA-IR	-0.064	0.746
HOMA2-%β *	0.198	0.181
CPR index *	0.058	0.689
HOMA2-%S *	0.231	0.118
QUICKI *	0.158	0.346
HbA1c (%, mmol/mol) *	-0.157	0.305
Total cholesterol (mg/dL)	-0.336	0.016
LDL cholesterol (mg/dL)	-0.342	0.014
HDL cholesterol (mg/dL) *	0.237	0.094
Triglycerides (mg/dL) *	-0.273	0.053
AST (IU/L) *	-0.038	0.790
ALT (IU/L) *	-0.031	0.830
γGTP (IU/L) *	-0.287	0.042
FIB-4 index *	-0.089	0.546
eGFR (mL/min)	-0.080	0.575
Urine albumin/Cre (mg/gCre) *	0.020	0.891

AST, Aspartate aminotransferase; ALT, Alanine aminotransferase; BMI, Body mass index; CPR index, C-peptide immunoreactivity index; eGFR, Estimated glomerular filtration rate; FIB-4 index, fibrosis index based on four factors; γGTP, γ-glutamyl transpeptidase; HOMA-IR, Homeostasis model assessment of insulin resistance; HOMA2-%β, Homeostasis model assessment for β-cell function; HOMA2-%S, Homeostasis model assessment for insulin sensitivity; HDL cholesterol, High-density lipoprotein cholesterol; LDL cholesterol, Low-density lipoprotein cholesterol; NGT, Normal glucose tolerance; T2DM, Type 2 diabetes; QUICKI, Quantitative insulin sensitivity check index

Univariate linear regression analyses were performed to evaluate the correlation between serum SerpinB1 levels and metabolic parameters.

* Variables and serum serpinB1 levels were log-transformed.

After adjusting for age and sex (Model 1), the serum SerpinB1 levels were positively correlated with HOMA2-%S (β = 0.319, *P* = 0.036), and negatively correlated with FBG (β = -0.365, *P* = 0.010), T-Chol (β = -0.396, *P* = 0.006), LDL-Chol (β = -0.411, *P* = 0.004), TG (β = -0.321, *P* = 0.026), and γGTP (β = -0.322, *P* = 0.026) in the T2DM subjects (**Table 3**). Furthermore, the adjustment for age, sex, and BMI (Model 2) strengthened the positive association between serum SerpinB1 levels and insulin sensitivity indices that were HOMA2%-S (β = 0.417, *P* = 0.010) and QUICKI (β = 0.519, *P* = 0.049). The negative correlation with FBG (β = -0.358, *P* = 0.012), T-Chol (β = -0.398, *P* = 0.006), LDL-Chol (β = -0.412, *P* = 0.004), TG (β = -0.428, *P* = 0.006), and γGTP (β = -0.364, *P* = 0.014) still remained in Model 2 (**Table 3**). No correlations were observed between SerpinB1 and other metabolic parameters, such as obesity and insulin secretion capacity or renal function (**Table 3**).

**Table 3:** Multivariate linear regression analyses for serum SerpinB1 levels in T2DM subjects.

	Model 1		Model 2	
	β	*P*	β	*P*
Estimated duration of diabetes (years) *	0.124	0.438	0.115	0.480
BMI *	0.112	0.485	-	-
Waist circumference (cm) *	0.119	0.942	-0.570	0.141
Systolic blood pressure (mmHg)	0.022	0.882	0.015	0.918
Diastolic blood pressure (mmHg)	-0.178	0.218	-0.187	0.200
Fasting blood glucose (mg/dL) *	-0.365	0.010	-0.358	0.012
Fasting serum insulin (μIU/mL) *	-0.161	0.454	-0.299	0.287
C-peptide (ng/mL) *	-0.158	0.287	-0.277	0.101
HOMA-IR	-0.243	0.275	-0.395	0.154
HOMA2-%β *	0.162	0.286	0.141	0.386
CPR index *	0.019	0.900	-0.044	0.794
HOMA2-%S *	0.319	0.036	0.417	0.010
QUICKI *	0.296	0.149	0.519	0.049
HbA1c (%, mmol/mol) *	-0.232	0.151	-0.230	0.157
Total cholesterol (mg/dL)	-0.396	0.006	-0.398	0.006
LDL cholesterol (mg/dL)	-0.411	0.004	-0.412	0.004
HDL cholesterol (mg/dL) *	0.251	0.078	0.283	0.052
Triglycerides (mg/dL) *	-0.321	0.026	-0.428	0.006
AST (IU/L) *	-0.083	0.574	-0.110	0.469
ALT (IU/L) *	-0.127	0.420	-0.152	0.346
γGTP (IU/L) *	-0.322	0.026	-0.364	0.014
FIB-4 index *	-0.012	0.946	-0.016	0.925
eGFR (mL/min)	-0.227	0.169	-0.232	0.241
Urine albumin/Cre (mg/gCre) *	0.049	0.736	0.036	0.809

AST, Aspartate aminotransferase; ALT, Alanine aminotransferase; BMI, Body mass index; CPR index, C-peptide immunoreactivity index; eGFR, Estimated glomerular filtration rate; FIB-4 index, fibrosis index based on four factors; γGTP, γ-glutamyl transpeptidase; HOMA-IR, Homeostasis model assessment of insulin resistance; HOMA2-%β, Homeostasis model assessment for β-cell function; HOMA2-%S, Homeostasis model assessment for insulin sensitivity; HDL cholesterol, High-density lipoprotein cholesterol; LDL cholesterol, Low-density lipoprotein cholesterol; NGT, Normal glucose tolerance; T2DM, Type 2 diabetes; QUICKI, Quantitative insulin sensitivity check index

Multivariate linear regression analyses adjusted for age and sex (Model 1) and age, sex, and BMI (Model 2) were performed to evaluate the correlation between serum SerpinB1 levels and metabolic parameters.

* Variables and serum serpinB1 levels were log-transformed.

We also analyzed by excluding subjects who were treated with insulin sensitizers, insulin secretagogues, or insulin (**S3-S6 Tables in S1 File**). In subjects with T2DM who were not using insulin, the serum SerpinB1 levels were negatively correlated with T-Chol (β = -0.603, *P* <0.001), LDL-Chol (β = -0.590, *P* = 0.001), and γGTP (β = -0.387, *P* = 0.043), and tended to be associated with higher HOMA2-%S (β = 0.343, *P* = 0.095) after adjusting for age and sex (Model 1) (**S4 Table in S1 File**). Moreover, in Model 2 (adjustment for age, sex, and BMI), the association with T-Chol (β = -0.610, *P* = 0.001), LDL-Chol (β = -0.591, *P* = 0.001), and γGTP (β = -0.554, *P* = 0.014) still remained significant, and HOMA2-%S (β = 0.811, *P* = 0.007), QUICKI (β = 0.519, *P* = 0.049), and TG (β = -0.483, *P* = 0.034) were revealed to have a correlation with serum SerpinB1 levels (**S4 Table in S1 File**). In subjects with T2DM not taking insulin sensitizers (metformin/thiazolidinediones), the serum SerpinB1 levels were positively correlated with QUICKI (β = 0.465, *P* = 0.047), and negatively correlated with T-Chol (β = -0.449, *P* = 0.012), LDL-Chol (β = -0.550, *P* = 0.002), and γGTP (β = -0.374, *P* = 0.039) after adjusting for age and sex (Model 1). These correlations were recapitulated after the adjustment for BMI as well as age and sex (Model 2, QUICKI: β = 0.689, *P* = 0.032, T-Chol: β = -0.449, *P* = 0.014, LDL-Chol: β = -0.550, *P* = 0.002, and γGTP: β = -0.396, *P* = 0.036) (**S5 Table in S1 File**). Additionally, HDL-Chol and TG exhibited significant correlations with serum SerpinB1 levels (HDL-Chol: β = 0.383, *P* = 0.049, and TG: β = -0.439, *P* = 0.036) (**S5 Table in S1 File**). In subjects with T2DM not taking insulin secretagogues (sulfonylureas/glinides), the serum SerpinB1 levels were negatively correlated with FBG (β = -0.449, *P* = 0.007), and tended to be associated with higher HOMA2-%S (β = 0.345, *P* = 0.051) after adjusting for age and sex (**S6 Table in S1 File**). In Model 2 (adjustment for age, sex, and BMI), there were the significant correlations between serum SerpinB1 levels and several parameters including waist circumference (β = -1.076, *P* = 0.018), FBG (β = -0.441, *P* = 0.011), and QUICKI (β = 0.813, *P* = 0.023) (**S6 Table in S1 File**). Thus, the trends of association were similar regardless of whether the T2DM subjects were treated with any anti-diabetes drugs or not.

## Discussion

In this study, we examined circulating SerpinB1 levels in Japanese subjects with T2DM or NGT. There was no difference in serum SerpinB1 levels between the 2 groups. In T2DM subjects, the serum SerpinB1 levels were positively correlated with HOMA2-%S, and negatively correlated with FBG, T-Chol, LDL-Chol, TG, and γGTP.

SerpinB1 was identified as a hepatokine that promoted pancreatic β-cell proliferation to compensate for insulin resistance in the liver-specific insulin receptor knockout mouse. Forkhead box protein O1 (FoxO1), a major transcription factor of insulin signaling in many organs, regulates SerpinB1 transcription in the liver through non-cell-autonomous factors from other tissues or cells in vivo [12, 13]. In our present study, SerpinB1 was positively associated with insulin sensitivity index and better blood glucose level, and negatively correlated with hyperlipidemia and hyper-concentration of γGTP, which were related to hepatic insulin resistance. In addition to insulin, other many factors, including glucagon, free fatty acid (FFA), nutrition, inflammation, and oxidative stress, also regulate FoxO1 activity through its expression, phosphorylation, or acetylation in the liver. Subjects with diabetes often exhibit hyperinsulinemia, glucagon dysregulation, hyperglycemia-induced microinflammation, oxidative stress, endoplasmic reticulum (ER) stress, and/or dyslipidemia, which might regulate the SerpinB1 expression via hepatic regulation of FoxO1. Further study is required to clarify which factor is involved in the production of SerpinB1 in Japanese subjects with T2DM. Moreover, Japanese individuals are thought to have less β-cell mass and be less obese [14], which results in reduced insulin secretion without severe insulin resistance compared to Caucasian subjects. Indeed, in this study, insulin resistance was not severe in T2DM subjects, which may have resulted in relatively low serum SerpinB1 levels compared to those in previous reports in the US [2, 4]. The low degree of insulin resistance in subjects with T2DM may enhance the influence of other factors that correlate with insulin sensitivity on SerpinB1 levels.

In this study, no correlation was observed between serum SerpinB1 and β-cell function, such as the CPR index or HOMA2-%β. Since Asians have less insulin secretion capacity than Caucasians [14], the effects of SerpinB1 or other growth factors on the regulation of β-cell mass might differ among ethnicities. Otherwise, the action of SerpinB1 on β-cells might be distorted in subjects with T2DM, which is accompanied by a decrease in β-cell mass.

To assess the impacts of anti-diabetes agents, we excluded T2DM subjects who were treated with insulin sensitizers, insulin secretagogues, or insulin. Even after excluding those subjects, there remains a tendency of the positive correlation between serum SerpinB1 and HOMA2-%S or QUICKI, and no association of SerpinB1 with HOMA2-%β or CPR index. Therefore, the correlation between SerpinB1 and insulin sensitivity or β-cell function in this study did not seem to be influenced by the use of anti-diabetes drugs.

Our study has several limitations. The main limitation is relatively small sample size of both NGT and T2DM subjects. To confirm the association between serum SerpinB1 levels and associated factors including insulin sensitivity indices, further analysis of drug-naïve subjects with T2DM is needed. This study is a cross-sectional design, which does not allow determination of causal direction of the results.

In conclusion, in Japanese T2DM subjects with less severe obesity, serum SerpinB1 is positively correlated with insulin sensitivity and better blood glucose level.

## Supporting information

S1 File (DOCX)
