# Supplementary material for: Association between circulating SerpinB1 levels and insulin sensitivity in Japanese with type 2 diabetes: A single-center, cross-sectional, observational study
Source: PLoS One. 2022 Nov 4;17(11):e0276915. doi: 10.1371/journal.pone.0276915 (PMC9635728; doi:10.1371/journal.pone.0276915)
Supplement: S1 File — (DOCX) [file pone.0276915.s001.docx]

**S1 Table. Use of anti-diabetic medicine in T2DM subjects.**

| Anti-diabetic medicine |  | No. subjects (%) |
| --- | --- | --- |
| Metformin |  | 19 (37.2) |
| Sulfonylureas |  | 13 (25.5) |
| Glinides |  | 1 (1.9) |
| Thiazolidinediones |  | 3 (5.8) |
| DPP-4 inhibitors |  | 19 (37.2) |
| α-Glucosidase inhibitors |  | 9 (17.6) |
| SGLT-2 inhibitors |  | 4 (7.8) |
| Insulin |  | 22 (43.1) |
| GLP-1 receptor agonists |  | 3 (5.8) |

T2DM, Type 2 diabetes; DPP-4, Dipeptidyl peptidase-4; SGLT-2, Sodium-glucose co-transporter-2; GLP-1, Glucagon-like peptide-1

**S2 Table. Serum SerpinB1 levels in NGT and T2DM subjects who were divided into quartiles according to HbA1c levels.**

|  |  | Serum SerpinB1 (ng/mL) |  | *P* value  (*vs.* NGT) | *P* value  (*vs.* Q1) | *P* value  (*vs.* Q2) | *P* value  (*vs.* Q3) |  |  |
| --- | --- | --- | --- | --- | --- | --- | --- | --- | --- |
| NGT (n = 12) |  | 1.8 ± 1.7 |  | - | - | - | - |  |  |
| Q1 (n = 11) |  | 1.4 ± 1.3 |  | 0.479 | - | - | - |  |  |
| Q2 (n = 10) |  | 1.6 ± 0.8 |  | 0.726 | 0.614 | - | - |  |  |
| Q3 (n = 12) |  | 1.3 ± 1.0 |  | 0.333 | 0.824 | 0.389 | - |  |  |
| Q4 (n = 12) |  | 1.1 ± 0.8 |  | 0.170 | 0.487 | 0.126 | 0.587 |  |  |

NGT, Normal glucose tolerance; T2DM, Type 2 diabetes

Serum SerpinB1 levels were categorized according to the quartile of HbA1c levels (first quartile (Q1): HbA1c ≤7.5%, second quartile (Q2): 7.5% < HbA1c ≤8.6%, third quartile (Q 3): 8.6% < HbA1c <10.3%, and fourth quartile (Q4): 10.3% ≤ HbA1c).

*P* values are the results of Student’s t-test. Data are shown as mean ± SD.

**S3 Table. Serum SerpinB1 levels and metabolic characteristics of T2DM subjects not treated with insulin, insulin sensitizers (metformin/** **thiazolidinediones), or insulin secretagogues (sulfonylureas/glinides).**

|  |  | T2DM  not using insulin |  |  | T2DM  not taking insulin sensitizers |  |  | T2DM  not taking insulin secretagogues |
| --- | --- | --- | --- | --- | --- | --- | --- | --- |
|  |  | value (n) |  |  | value (n) |  |  | value (n) |
| Serum SerpinB1 (ng/mL) |  | 1.5 ± 1.1 (29) |  |  | 1.3 ± 1.1 (32) |  |  | 1.3 ± 1.0 (34) |
| Age (years) |  | 54.5 ± 12.3 (29) |  |  | 55.3 ± 12.9 (32) |  |  | 54.9 ± 12.3 (34) |
| No. men/women |  | 18/11 |  |  | 19/13 |  |  | 18/16 |
| Estimated duration of diabetes (years) |  | 7.2 ± 6.8 (24) |  |  | 9.2 ± 8.2 (25) |  |  | 7.9 ± 8.4 (24) |
| BMI |  | 27.3 ± 7.3 (29) |  |  | 27.1 ± 6.4 (32) |  |  | 26.8 ± 6.2 (34) |
| Waist circumference (cm) |  | 94.4 ± 14.5 (28) |  |  | 94.5 ± 13.8 (30) |  |  | 93.8 ± 13.0 (32) |
| Systolic blood pressure (mmHg) |  | 134.9 ± 21.9 (29) |  |  | 130.9 ± 18.0 (32) |  |  | 134.7 ± 18.6 (34) |
| Diastolic blood pressure (mmHg) |  | 82.6 ± 17.7 (29) |  |  | 81.9 ± 15.7 (32) |  |  | 79.9 ± 15.1 (34) |
| Fasting blood glucose (mg/dL) |  | 162.9 ± 53.4 (28) |  |  | 162.5 ± 60.3 (31) |  |  | 164.1 ± 54.6 (34) |
| Fasting serum insulin (μIU/mL) |  | 12.6 ± 9.4 (28) |  |  | 12.5 ± 8.1 (18) |  |  | 10.0 ± 5.8 (17) |
| C-peptide (ng/mL) |  | 3.1 ± 1.6 (28) |  |  | 2.7 ± 1.4 (31) |  |  | 2.2 ± 1.2 (34) |
| HOMA-IR |  | 4.8 ± 3.1 (28) |  |  | 4.8 ± 3.1 (18) |  |  | 3.6 ± 2.2 (17) |
| HOMA2-%β |  | 76.4 ± 66.8 (27) |  |  | 53.6 ± 36.3 (18) |  |  | 56.8 ± 32.9 (32) |
| CPR index |  | 2.2 ± 1.7 (28) |  |  | 1.8 ± 1.1 (31) |  |  | 1.5 ± 0.9 (34) |
| HOMA2-%S |  | 45.0 ± 26.2 (27) |  |  | 86.6 ± 66.9 (18) |  |  | 62.2 ± 37.5 (32) |
| QUICKI |  | 0.3 ± 0.0 (28) |  |  | 0.3 ± 0.0 (18) |  |  | 0.3 ± 0.0 (17) |
| HbA1c (%) |  | 9.0 ± 2.3 (26) |  |  | 9.2 ± 2.5 (28) |  |  | 9.2 ± 2.7 (32) |
| HbA1c (mmol/mol) |  | 74.6 ± 25.1 (26) |  |  | 76.5 ± 27.6 (28) |  |  | 77.0 ± 29.0 (32) |
| Total cholesterol (mg/dL) |  | 196.8 ± 44.5 (29) |  |  | 204.0 ± 43.6 (32) |  |  | 194.1 ± 36.7 (34) |
| LDL cholesterol (mg/dL) |  | 118.2 ± 36.8 (29) |  |  | 123.3 ± 35.4 (32) |  |  | 115.6 ± 31.2 (34) |
| HDL cholesterol (mg/dL) |  | 64.7 ± 73.7 (29) |  |  | 50.1 ± 16.3 (32) |  |  | 52.4 ± 15.6 (34) |
| Triglycerides (mg/dL) |  | 168.2 ± 110.0 (29) |  |  | 191.4 ± 113.1 (32) |  |  | 176.3 ± 106.9 (34) |
| AST (IU/L) |  | 27.7 ± 20.8 (29) |  |  | 33.7 ± 40.7 (32) |  |  | 28.8 ± 38.4 (34) |
| ALT (IU/L) |  | 33.6 ± 28.9 (29) |  |  | 37.5 ± 36.1 (32) |  |  | 28.6 ± 24.0 (34) |
| γGTP (IU/L) |  | 66.7 ± 93.6 (29) |  |  | 96.7 ± 188.8 (32) |  |  | 72.3 ± 171.5 (34) |
| FIB-4 index |  | 1.4 ± 1.0 (27) |  |  | 1.8 ± 3.0 (30) |  |  | 1.7 ± 2.9 (33) |
| eGFR (mL/min) |  | 80.0 ± 24.2 (29) |  |  | 79.0 ± 28.8 (32) |  |  | 81.2 ± 28.0 (34) |
| Urine albumin/Cre (mg/gCre) |  | 163.4 ± 590.6 (29) |  |  | 182.5 ± 618.5 (32) |  |  | 183.7 ± 601.8 (34) |

AST, Aspartate aminotransferase; ALT, Alanine aminotransferase; BMI, Body mass index; CPR index, C-peptide immunoreactivity index; eGFR, Estimated glomerular filtration rate; FIB-4 index, fibrosis index based on four factors; γGTP, γ-glutamyl transpeptidase; HOMA-IR, Homeostasis model assessment of insulin resistance; HOMA2-%β, Homeostasis model assessment for β-cell function; HOMA2-%S, Homeostasis model assessment for insulin sensitivity; HDL cholesterol, High-density lipoprotein cholesterol; LDL cholesterol, Low-density lipoprotein cholesterol; NGT, Normal glucose tolerance; T2DM, Type 2 diabetes; QUICKI, Quantitative insulin sensitivity check index

Data are shown as mean ± SD.

**S4 Table. Univariate and multivariate linear regression analyses for serum SerpinB1 levels in T2DM subjects not treated with insulin.**

|  |  | Univariate | |  |  | Multivariate (Model 1) | |  |  | Multivariate (Model 2) | |
| --- | --- | --- | --- | --- | --- | --- | --- | --- | --- | --- | --- |
|  |  | β | *P* |  |  | β | *P* |  |  | β | *P* |
| Age (years) |  | -0.268 | 0.159 |  |  | - | - |  |  | - | - |
| Sex |  | 0.024 | 0.902 |  |  | - | - |  |  | - | - |
| Estimated duration of diabetes (years) |  | 0.048 | 0.825 |  |  | 0.020 | 0.461 |  |  | 0.474 | 0.927 |
| BMI * |  | 0.119 | 0.540 |  |  | 0.021 | 0.925 |  |  | - | - |
| Waist circumference (cm) * |  | -0.025 | 0.899 |  |  | -0.100 | 0.641 |  |  | -0.643 | 0.218 |
| Systolic blood pressure (mmHg) |  | 0.054 | 0.781 |  |  | 0.024 | 0.903 |  |  | 0.021 | 0.917 |
| Diastolic blood pressure (mmHg) |  | -0.182 | 0.346 |  |  | -0.180 | 0.355 |  |  | -0.188 | 0.351 |
| Fasting blood glucose (mg/dL) |  | -0.291 | 0.133 |  |  | -0.304 | 0.118 |  |  | -0.307 | 0.122 |
| Fasting serum insulin (μIU/mL) * |  | -0.049 | 0.804 |  |  | -0.161 | 0.454 |  |  | -0.299 | 0.287 |
| C-peptide (ng/mL) |  | -0.029 | 0.884 |  |  | -0.145 | 0.502 |  |  | -0.396 | 0.237 |
| HOMA-IR |  | -0.064 | 0.746 |  |  | -0.243 | 0.275 |  |  | -0.395 | 0.154 |
| HOMA2-%β * |  | 0.185 | 0.354 |  |  | 0.159 | 0.447 |  |  | 0.172 | 0.449 |
| CPR index * |  | 0.072 | 0.715 |  |  | 0.012 | 0.955 |  |  | -0.007 | 0.978 |
| HOMA2-%S * |  | 0.239 | 0.229 |  |  | 0.343 | 0.095 |  |  | 0.811 | 0.007 |
| QUICKI * |  | 0.185 | 0.346 |  |  | 0.296 | 0.149 |  |  | 0.519 | 0.049 |
| HbA1c (%, mmol/mol) |  | -0.157 | 0.444 |  |  | -0.211 | 0.314 |  |  | -0.223 | 0.308 |
| Total cholesterol (mg/dL) |  | -0.551 | 0.002 |  |  | -0.603 | <0.001 |  |  | -0.610 | 0.001 |
| LDL cholesterol (mg/dL) |  | -0.541 | 0.003 |  |  | -0.590 | 0.001 |  |  | -0.591 | 0.001 |
| HDL cholesterol (mg/dL) * |  | 0.208 | 0.278 |  |  | 0.219 | 0.256 |  |  | 0.249 | 0.229 |
| Triglycerides (mg/dL) * |  | -0.313 | 0.099 |  |  | -0.338 | 0.079 |  |  | -0.483 | 0.034 |
| AST (IU/L) * |  | 0.008 | 0.967 |  |  | -0.139 | 0.534 |  |  | -0.213 | 0.432 |
| ALT (IU/L) * |  | 0.023 | 0.904 |  |  | -0.138 | 0.535 |  |  | -0.175 | 0.480 |
| γGTP (IU/L) * |  | -0.315 | 0.097 |  |  | -0.387 | 0.043 |  |  | -0.554 | 0.014 |
| FIB-4 index * |  | -0.081 | 0.687 |  |  | 0.059 | 0.824 |  |  | 0.056 | 0.841 |
| eGFR (mL/min) |  | -0.043 | 0.827 |  |  | -0.167 | 0.427 |  |  | -0.226 | 0.381 |
| Urine albumin/Cre (mg/gCre) * |  | -0.016 | 0.934 |  |  | -0.013 | 0.947 |  |  | -0.014 | 0.944 |

AST, Aspartate aminotransferase; ALT, Alanine aminotransferase; BMI, Body mass index; CPR index, C-peptide immunoreactivity index; eGFR, Estimated glomerular filtration rate; FIB-4 index, fibrosis index based on four factors; γGTP, γ-glutamyl transpeptidase; HOMA-IR, Homeostasis model assessment of insulin resistance; HOMA2-%β, Homeostasis model assessment for β-cell function; HOMA2-%S, Homeostasis model assessment for insulin sensitivity; HDL cholesterol, High-density lipoprotein cholesterol; LDL cholesterol, Low-density lipoprotein cholesterol; NGT, Normal glucose tolerance; T2DM, Type 2 diabetes; QUICKI, Quantitative insulin sensitivity check index

Univariate and multivariate linear regression analyses adjusted for age and sex (Model 1), and age, sex, and BMI (Model 2) were performed to evaluate the correlation between serum SerpinB1 levels and metabolic parameters.

* Variables and serum SerpinB1 levels were log-transformed.

**S5 Table. Univariate and multivariate linear regression analyses for serum SerpinB1 levels in T2DM subjects not treated with insulin sensitizers (metformin/** **thiazolidinediones).**

|  |  | Univariate | |  |  | Multivariate (Model 1) | |  |  | Multivariate (Model 2) | |
| --- | --- | --- | --- | --- | --- | --- | --- | --- | --- | --- | --- |
|  |  | β | *P* |  |  | β | *P* |  |  | β | *P* |
| Age (years) |  | -0.325 | 0.069 |  |  | - | - |  |  | - | - |
| sex |  | -0.034 | 0.854 |  |  | - | - |  |  | - | - |
| Estimated duration of diabetes (years) |  | 0.061 | 0.774 |  |  | 0.087 | 0.672 |  |  | 0.080 | 0.708 |
| BMI * |  | 0.079 | 0.669 |  |  | 0.014 | 0.942 |  |  | - | - |
| Waist circumference (cm) * |  | -0.082 | 0.666 |  |  | -0.093 | 0.640 |  |  | -0.864 | 0.116 |
| Systolic blood pressure (mmHg) |  | 0.079 | 0.666 |  |  | 0.055 | 0.763 |  |  | 0.055 | 0.773 |
| Diastolic blood pressure (mmHg) |  | -0.072 | 0.694 |  |  | -0.104 | 0.577 |  |  | -0.109 | 0.571 |
| Fasting blood glucose (mg/dL) |  | -0.277 | 0.134 |  |  | -0.251 | 0.168 |  |  | -0.251 | 0.178 |
| Fasting serum insulin (μIU/mL) |  | -0.053 | 0.834 |  |  | -0.327 | 0.237 |  |  | -0.321 | 0.313 |
| C-peptide (ng/mL) |  | -0.147 | 0.430 |  |  | -0.289 | 0.125 |  |  | -0.383 | 0.074 |
| HOMA-IR |  | -0.121 | 0.633 |  |  | -0.411 | 0.126 |  |  | -0.443 | 0.164 |
| HOMA2-%β |  | 0.090 | 0.721 |  |  | -0.014 | 0.959 |  |  | 0.006 | 0.983 |
| CPR index * |  | -0.009 | 0.963 |  |  | -0.122 | 0.518 |  |  | -0.152 | 0.461 |
| HOMA2-%S * |  | 0.248 | 0.322 |  |  | 0.405 | 0.102 |  |  | 0.481 | 0.121 |
| QUICKI |  | 0.357 | 0.146 |  |  | 0.465 | 0.047 |  |  | 0.689 | 0.032 |
| HbA1c (%, mmol/mol) |  | -0.062 | 0.755 |  |  | -0.193 | 0.337 |  |  | -0.196 | 0.341 |
| Total cholesterol (mg/dL) |  | -0.342 | 0.055 |  |  | -0.449 | 0.012 |  |  | -0.449 | 0.014 |
| LDL cholesterol (mg/dL) |  | -0.380 | 0.032 |  |  | -0.550 | 0.002 |  |  | -0.550 | 0.002 |
| HDL cholesterol (mg/dL) * |  | 0.253 | 0.163 |  |  | -0.333 | 0.065 |  |  | 0.383 | 0.049 |
| Triglycerides (mg/dL) * |  | -0.255 | 0.178 |  |  | -0.337 | 0.066 |  |  | -0.439 | 0.036 |
| AST (IU/L) * |  | -0.006 | 0.974 |  |  | -0.145 | 0.450 |  |  | -0.159 | 0.431 |
| ALT (IU/L) * |  | 0.041 | 0.824 |  |  | -0.177 | 0.396 |  |  | -0.195 | 0.377 |
| γGTP (IU/L) * |  | -0.284 | 0.115 |  |  | -0.374 | 0.039 |  |  | -0.396 | 0.036 |
| FIB-4 index * |  | -0.162 | 0.392 |  |  | -0.015 | 0.946 |  |  | -0.012 | 0.956 |
| eGFR (mL/min) |  | -0.034 | 0.853 |  |  | -0.278 | 0.189 |  |  | -0.374 | 0.138 |
| Urine albumin/Cre (mg/gCre) * |  | 0.054 | 0.769 |  |  | 0.122 | 0.510 |  |  | 0.130 | 0.512 |

AST, Aspartate aminotransferase; ALT, Alanine aminotransferase; BMI, Body mass index; CPR index, C-peptide immunoreactivity index; eGFR, Estimated glomerular filtration rate; FIB-4 index, fibrosis index based on four factors; γGTP, γ-glutamyl transpeptidase; HOMA-IR, Homeostasis model assessment of insulin resistance; HOMA2-%β, Homeostasis model assessment for β-cell function; HOMA2-%S, Homeostasis model assessment for insulin sensitivity; HDL cholesterol, High-density lipoprotein cholesterol; LDL cholesterol, Low-density lipoprotein cholesterol; NGT, Normal glucose tolerance; T2DM, Type 2 diabetes; QUICKI, Quantitative insulin sensitivity check index

Univariate and multivariate linear regression analyses adjusted for age and sex (Model 1), and age, sex, and BMI (Model 2) were performed to evaluate the correlation between serum SerpinB1 levels and metabolic parameters.

* Variables and serum SerpinB1 levels were log-transformed.

**S6 Table. Univariate and multivariate linear regression analyses for serum SerpinB1 levels in T2DM subjects not treated with insulin secretagogues (sulfonylureas/glinides).**

|  |  | Univariate | |  |  | Multivariate (Model 1) | |  |  | Multivariate (Model 2) | |
| --- | --- | --- | --- | --- | --- | --- | --- | --- | --- | --- | --- |
|  |  | β | *P* |  |  | β | *P* |  |  | β | *P* |
| Age (years) |  | -0.185 | 0.273 |  |  | - | - |  |  | - | - |
| sex |  | 0.032 | 0.852 |  |  | - | - |  |  | - | - |
| Estimated duration of diabetes (years) |  | 0.218 | 0.266 |  |  | 0.203 | 0.329 |  |  | 0.225 | 0.308 |
| BMI * |  | 0.097 | 0.566 |  |  | 0.078 | 0.669 |  |  | - | - |
| Waist circumference (cm) * |  | -0.037 | 0.834 |  |  | -0.054 | 0.772 |  |  | -1.076 | 0.018 |
| Systolic blood pressure (mmHg) |  | -0.167 | 0.322 |  |  | -0.160 | 0.351 |  |  | -0.213 | 0.237 |
| Diastolic blood pressure (mmHg) |  | -0.224 | 0.183 |  |  | -0.242 | 0.168 |  |  | -0.268 | 0.152 |
| Fasting blood glucose (mg/dL) * |  | -0.417 | 0.010 |  |  | -0.449 | 0.007 |  |  | -0.441 | 0.011 |
| Fasting serum insulin (μIU/mL) |  | -0.027 | 0.916 |  |  | -0.126 | 0.632 |  |  | -0.155 | 0.651 |
| C-peptide (ng/mL) |  | -0.092 | 0.587 |  |  | -0.103 | 0.559 |  |  | -0.076 | 0.713 |
| HOMA-IR |  | -0.171 | 0.512 |  |  | -0.363 | 0.197 |  |  | -0.540 | 0.148 |
| HOMA2-%β * |  | 0.250 | 0.148 |  |  | 0.245 | 0.159 |  |  | 0.294 | 0.117 |
| CPR index * |  | 0.082 | 0.631 |  |  | 0.080 | 0.643 |  |  | 0.135 | 0.492 |
| HOMA2-%S * |  | 0.281 | 0.101 |  |  | 0.345 | 0.051 |  |  | 0.318 | 0.104 |
| QUICKI |  | 0.354 | 0.149 |  |  | 0.413 | 0.088 |  |  | 0.813 | 0.023 |
| HbA1c (%, mmol/mol) * |  | -0.146 | 0.403 |  |  | -0.224 | 0.233 |  |  | -0.208 | 0.308 |
| Total cholesterol (mg/dL) |  | 0.029 | 0.865 |  |  | -0.014 | 0.938 |  |  | 0.005 | 0.979 |
| LDL cholesterol (mg/dL) |  | -0.009 | 0.959 |  |  | -0.053 | 0.766 |  |  | -0.057 | 0.766 |
| HDL cholesterol (mg/dL) * |  | 0.283 | 0.090 |  |  | 0.273 | 0.107 |  |  | 0.297 | 0.118 |
| Triglycerides (mg/dL) * |  | -0.175 | 0.300 |  |  | -0.213 | 0.221 |  |  | -0.217 | 0.315 |
| AST (IU/L) * |  | 0.012 | 0.954 |  |  | -0.009 | 0.961 |  |  | 0.044 | 0.813 |
| ALT (IU/L) * |  | 0.004 | 0.984 |  |  | -0.059 | 0.745 |  |  | -0.018 | 0.927 |
| γGTP (IU/L) * |  | -0.242 | 0.149 |  |  | -0.245 | 0.172 |  |  | -0.182 | 0.350 |
| FIB-4 index * |  | -0.075 | 0.663 |  |  | 0.051 | 0.804 |  |  | 0.148 | 0.493 |
| eGFR (mL/min) |  | -0.024 | 0.889 |  |  | -0.199 | 0.345 |  |  | -0.325 | 0.224 |
| Urine albumin/Cre (mg/gCre) * |  | -0.002 | 0.991 |  |  | 0.027 | 0.877 |  |  | -0.077 | 0.690 |

AST, Aspartate aminotransferase; ALT, Alanine aminotransferase; BMI, Body mass index; CPR index, C-peptide immunoreactivity index; eGFR, Estimated glomerular filtration rate; FIB-4 index, fibrosis index based on four factors; γGTP, γ-glutamyl transpeptidase; HOMA-IR, Homeostasis model assessment of insulin resistance; HOMA2-%β, Homeostasis model assessment for β-cell function; HOMA2-%S, Homeostasis model assessment for insulin sensitivity; HDL cholesterol, High-density lipoprotein cholesterol; LDL cholesterol, Low-density lipoprotein cholesterol; NGT, Normal glucose tolerance; T2DM, Type 2 diabetes; QUICKI, Quantitative insulin sensitivity check index

Univariate and multivariate linear regression analyses adjusted for age and sex (Model 1), and age, sex, and BMI (Model 2) were performed to evaluate the correlation between serum SerpinB1 levels and metabolic parameters.

* Variables and serum SerpinB1 levels were log-transformed.
